# Supplementary material for: Calculating fast differential genome coverages among metagenomic sources using micov
Source: Commun Biol. 2025 Nov 20;8:1624. doi: 10.1038/s42003-025-09007-6 (PMC12635244; doi:10.1038/s42003-025-09007-6)
Supplement: Supplementary file 1 — Supplementary Material [file 42003_2025_9007_MOESM1_ESM.pdf]

SUPPLEMENTARY INFORMATION

a

	statistic	p-value	corrected
Country	112.53	1.00E-06	2.80E-05
PC351	74.97	1.00E-06	2.80E-05
Country_PC351	61.20	1.00E-06	2.80E-05
Bowel Movement Quality_PC351	10.75	1.00E-06	2.80E-05
Antibiotic History_PC351	9.86	1.00E-06	2.80E-05
Types of Plants_PC351	8.16	1.00E-06	2.80E-05
Bowel Movement Frequency_PC351	7.30	1.00E-06	2.80E-05
Bowel Movement Quality	3.91	3.10E-05	8.68E-04
Bowel Movement Frequency	2.72	2.23E-04	6.24E-03
Antibiotic History	2.46	4.16E-03	1.16E-01
Types of Plants	1.82	2.59E-02	7.25E-01

b

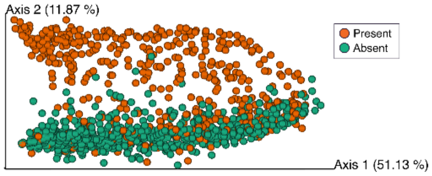

c

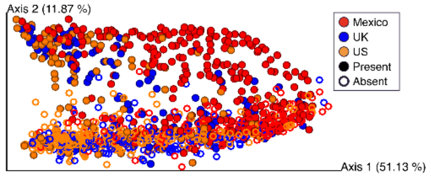

d

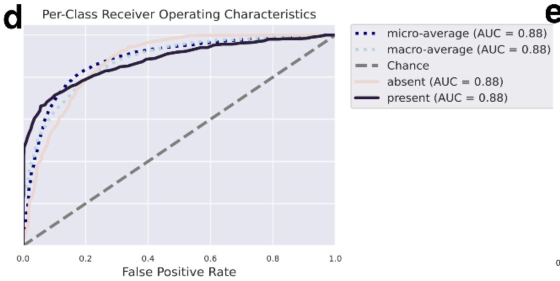

e

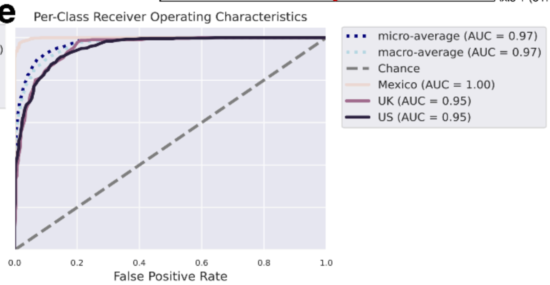

f

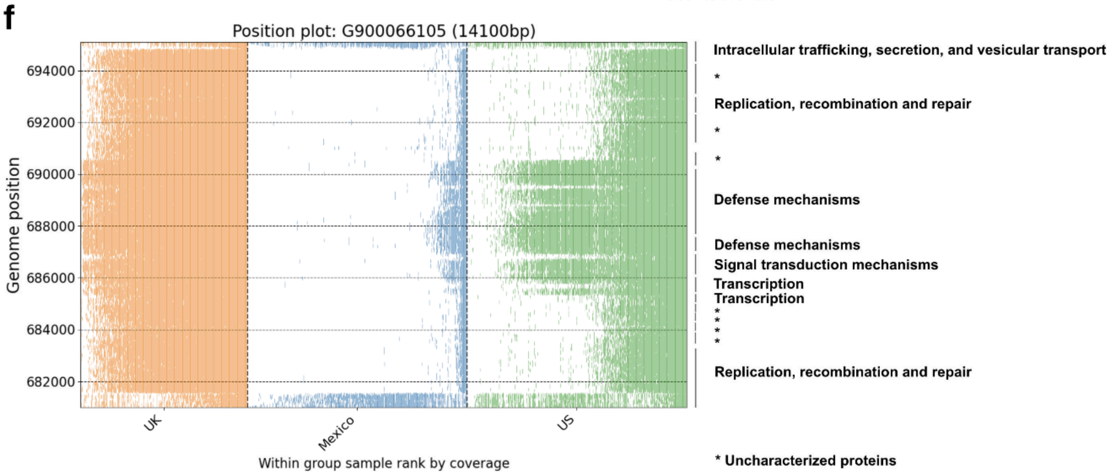

g

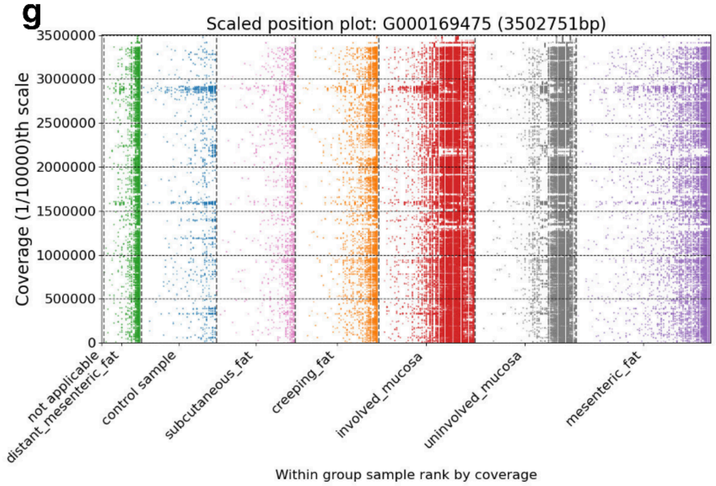

Supplementary Figure S1: (a) Weighted UniFrac PERMANOVA statistics of THDMI 16S rRNA V4 generated from the same physical specimens in Figure 1a-h, the full set of tests run is in Supplementary Table 2. (b-c) Principal coordinates of Weighted UniFrac distances from the THDMI 16S rRNA V4 data colored by (b) PC351 and (c) country. (d) A nested crossfold validated random forest classifier predicting the presence/absence of PC351 using the THDMI 16S rRNA V4 data. (e) A nested crossfold validated random forest classifier predicting the individual's country using the THDMI metagenomic data. (f) COG (Clusters of Orthologous Groups) annotations present in G900066105:682,000-695,000 (L682). (g) A scaled position plot of *M. gnavus* coverage in the visceral fat data.

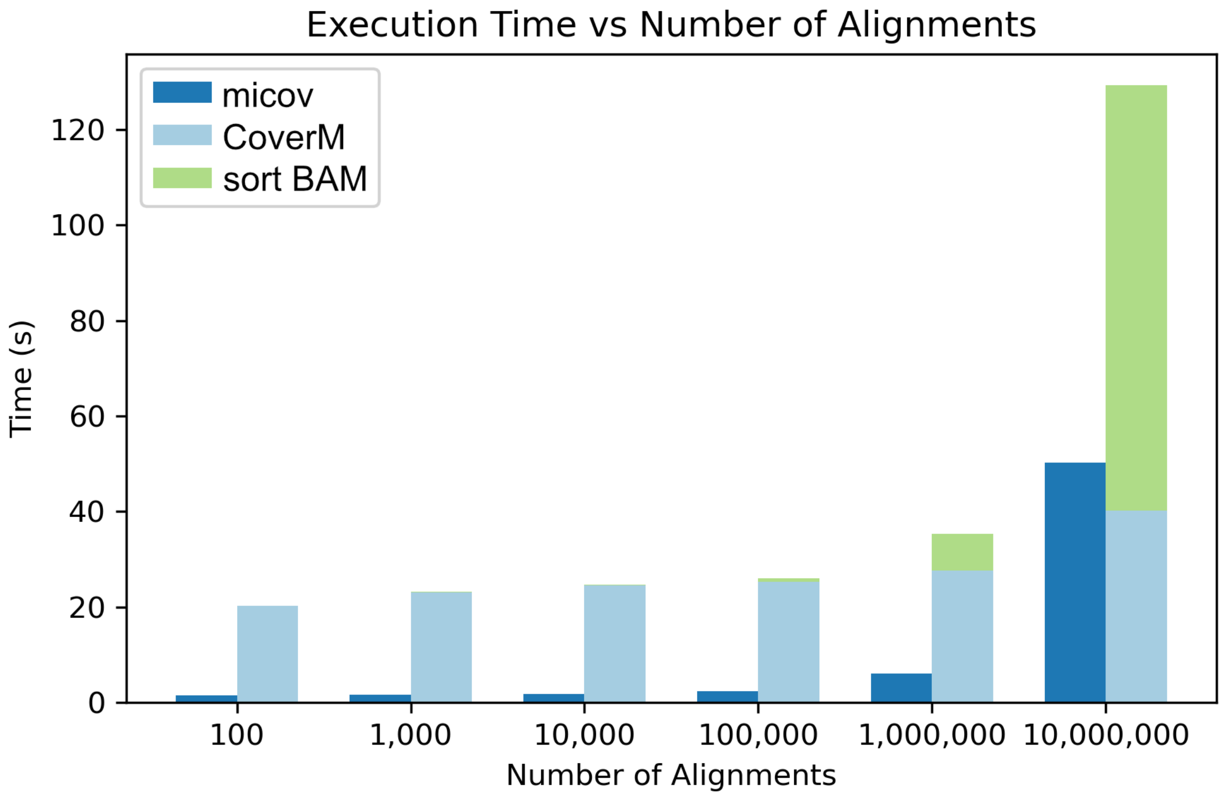

Supplementary Figure S2: Execution time of micov and CoverM for samples of 100 to 10 million alignments. Alignments were randomly sampled from the THDMI dataset. All tests were run using a single thread (*POLARS_MAX_THREADS*=1 for micov, *-t* 1 for CoverM) to avoid program-specific parallel overhead. CoverM requires sorted BAM files prior to coverage computation, while micov is agnostic to alignment sort order. To account for this difference, we included BAM sorting time as part of CoverM's total runtime cost. In contrast, micov runs directly on unsorted SAM files, simplifying the process. Across all input sizes, micov completed faster than CoverM when sorting time was included. For example, at 10 million alignments, micov completed in 50.3 seconds, while CoverM (including sorting) took 129.3 seconds - a 61% speedup. The coverage calculation step alone (excluding sorting) was faster for CoverM at the largest scale (40.2s v.s. 50.3s, Supplementary Table 3). We observed an increase in micov execution time from 1 million to 10 million alignments, indicating potential for improving scalability. However, despite this increase, micov completes the coverage calculations at a speed comparable to CoverM. Notably, micov outperformed CoverM for input sizes up to 1 million alignments. These results reasonably support a claim that micov is rapid, as the state-of-the-art tool CoverM describes itself as fast, and micov matches or exceeds its performance.

Algorithm 1: CIGAR Interpretation Algorithm

```
Data: cigar ;                                        /* a cigar string */
Result: a + o ;                      /* covered length of a single read */
a, o = 0 ;                            /* keep track of align and offset */
n = '' ;                                            /* current number */
for c in cigar do
    if c in 'MDIHNPSX=' then
        if c in 'MX=' then
            a += int(n)
        else if c in 'DN' then
            o += int(n)
        n = ''
    else
        n += c ;                               /* not a cigar operation */
    end
end
```

Supplementary Figure S3: micov pseudocode for the CIGAR interpretation algorithm

Supplementay Table 1. Functional annotations of *Lachnospiraceae* sp. 682,000-695,000 (L682)

ORF ID	Start Position	Stop Position	Gene Length	Uniref ID	Uniref Name	KO Name	KEGG Pathways Name	Pfam Name	Eggnog Seed Description	COG Category Name
G900066105_616	694999	695661	662	A0A173VCL8	Type IV secretion system protein VirD4	virD4, lvhD4; type IV secretion system protein VirD4 [EC:7.4.2.8	Bacterial secretion system	TraG-D_C	COG COG3505 Type IV secretory pathway, VirD4 components	Intracellular trafficking, secretion, and vesicular transport
G900066105_615	693022	694362	1340	A0A174PV93	Predicted P-loop ATPase and inactivated derivatives	NA	NA	VirE	Virulence-associated protein E	Function unknown
G900066105_614	692460	693080	620	A0A174PU06	DNA primase	dnaG; DNA primase [EC:2.7.7.101]	DNA replication	zf-CHC2	CHC2 zinc finger domain protein	Replication, recombination and repair
G900066105_613	691164	692399	1235	A0A174PPG1	Uncharacterized protein	NA	NA	NA	Psort location Cytoplasmic, score 8.96	Function unknown
G900066105_612	690338	690700	362	A0A1C6C598	Uncharacterized protein	NA	NA	NA	NA	NA
G900066105_611	687791	690295	2504	A0A329UNT6	ABC transporter permease	ABC.CD.P; putative ABC transport system permease protein	NA	MacB_PCD,FtsX,	FtsX-like permease family	Defense mechanisms
G900066105_610	687111	687791	680	A0A174PZK8	Lipoprotein-releasing system ATP-binding protein LolD	ABC.CD.A; putative ABC transport system ATP-binding protein	NA	ABC_tran	ABC transporter	Defense mechanisms
G900066105_609	686003	687010	1007	A0A174PPT8	Histidine kinase	NA	NA	HisKA,HATPase_c	Histidine kinase	Signal transduction mechanisms
G900066105_608	685330	686001	671	A0A174ZXH8	Stage 0 sporulation protein A homolog	NA	NA	Response_reg,Trans_reg_C	COG COG0745 Response regulators consisting of a CheY-like receiver domain and a winged-helix DNA-binding domain	Transcription
G900066105_607	685085	685333	248	A0A174PQ22	Predicted transcriptional regulator	putative transcriptional regulator	NA	HTH_26	Transcriptional regulator	Transcription
G900066105_606	684578	684868	290	A0A174PUW7	Uncharacterized protein	NA	NA	NA	NA	NA
G900066105_605	684307	684585	278	A0A174PYJ1	Uncharacterized protein	NA	NA	NA	NA	NA
G900066105_604	683903	684184	281	A0A174PQB8	Uncharacterized protein	NA	NA	NA	Psort location Cytoplasmic, score	Function unknown
G900066105_603	683504	683902	398	A0A174PXN6	Uncharacterized protein	NA	NA	TnpV	Transposon-encoded protein TnpV	Function unknown
G900066105_602	681639	683507	1868	A0A174PRC3	Recombinase	spoIVCA; site-specific DNA recombinase	NA	Resolvase, Recombinase,Zn_ribbon_recom,DUF4368	Recombinase	Replication, recombination and repair

Supplementary Table 2. Full PERMANOVA and KS test results
PC351- raw (Figure 2b)

**label_A**	**label_B**	**ks-statistic**	**ks-pvalue**	**corrected**
Mexico_abse	Mexico_pres	0.81366374	3.11E-79	4.67E-78
Mexico_abse	UK_absent	0.26846915	8.34E-07	1.25E-05
Mexico_abse	UK_present	0.80373832	1.71E-45	2.57E-44
Mexico_abse	US_absent	0.18016058	8.02E-04	1.20E-02
Mexico_abse	US_present	0.76521739	2.27E-42	3.41E-41
Mexico_pres	UK_absent	0.88590604	4.15E-100	6.23E-99
Mexico_pres	UK_present	0.31233143	2.62E-07	3.94E-06
Mexico_pres	US_absent	0.82879127	9.93E-105	1.49E-103
Mexico_pres	US_present	0.34654217	2.23E-09	3.35E-08
UK_absent	UK_present	0.85046729	4.94E-53	7.41E-52
UK_absent	US_absent	0.12442911	4.34E-02	6.51E-01
UK_absent	US_present	0.84735256	9.80E-55	1.47E-53
UK_present	US_absent	0.80971711	4.08E-53	6.12E-52
UK_present	US_present	0.16944332	7.11E-02	1.00E+00
US_absent	US_present	0.77894891	6.12E-51	9.18E-50

EPEC-raw (Figure 2h)

label_A	label_B	ks-statistic	ks-pvalue	corrected
0	0.1	0.63071895	2.47E-22	3.71E-21
0	1	0.59117647	5.43E-17	8.15E-16
0	10	0.67647059	5.59E-17	8.39E-16
0	100	0.64705882	2.25E-20	3.38E-19
0	1000	0.77777778	2.00E-14	3.00E-13
0.1	1	0.05962441	9.99E-01	1.00E+00
0.1	10	0.13682093	6.44E-01	1.00E+00
0.1	100	0.06230604	9.98E-01	1.00E+00
0.1	1000	0.40903756	3.50E-03	5.25E-02
1	10	0.11666667	8.46E-01	1.00E+00
1	100	0.06666667	9.97E-01	1.00E+00
1	1000	0.35833333	1.93E-02	2.90E-01
10	100	0.11824052	8.29E-01	1.00E+00
10	1000	0.29166667	1.22E-01	1.00E+00
100	1000	0.36511299	1.49E-02	2.24E-01

thdmi-wgs-weighted-raw (Figure 2c)

variable	statistic	p-value	corrected
wgs_with_prev	inf	1.00E+00	1.00E+00
wgs_with_prev_gt25	141.07118	1.00E-06	2.80E-05
16s_with_prev_gt25	123.1188	1.00E-06	2.80E-05
G000157935_351299_354812	78.66073	1.00E-06	2.80E-05
G000157935_1025794_1029307	71.82508	1.00E-06	2.80E-05
G000157935_189702_193215	70.6777	1.00E-06	2.80E-05
G000157935_1162801_1166314	69.626114	1.00E-06	2.80E-05
thdmi_cohort	63.19779	1.00E-06	2.80E-05
G000157935_2532868_2536381	57.603428	1.00E-06	2.80E-05
G000157935_1208470_1211983	51.671837	1.00E-06	2.80E-05
thdmi_cohort_G000157935_351299_354812	38.150265	1.00E-06	2.80E-05
G000157935_1647594_1651107	21.973078	1.00E-06	2.80E-05
G000157935_7026_10539	21.595299	1.00E-06	2.80E-05
G000157935_2817420_2820933	18.55071	1.00E-06	2.80E-05
G000157935_266987_270500	11.678018	4.00E-06	1.12E-04
16s_with_prev	10.765409	1.60E-05	4.48E-04
bowel_movement_quality_G000157935_351299_354812	10.596246	1.00E-06	2.80E-05
antibiotic_history_G000157935_351299_354812	10.384662	1.00E-06	2.80E-05
G000157935_270500_274013	9.014177	4.40E-05	1.23E-03
types_of_plants_G000157935_351299_354812	8.414935	1.00E-06	2.80E-05
bowel_movement_frequency_G000157935_351299_354812	7.346751	1.00E-06	2.80E-05
G000157935_1064437_1067950	4.7770505	3.47E-03	9.71E-02
bmi_cat	3.2265382	1.05E-03	2.95E-02
age_cat	3.1628249	3.40E-05	9.52E-04
bowel_movement_quality	2.9199111	8.44E-04	2.36E-02
antibiotic_history	2.4672356	3.78E-03	1.06E-01
bowel_movement_frequency	2.2055306	2.88E-03	8.06E-02
types_of_plants	1.4526967	9.80E-02	1.00E+00

thdmi-16s-weighted-raw (Figure S1a)

variable	statistic	p-value	corrected
wgs_with_prev	inf	1.00E+00	1.00E+00
16s_with_prev_gt25	163.34203	1.00E-06	2.80E-05
wgs_with_prev_gt25	141.88531	1.00E-06	2.80E-05
thdmi_cohort	112.52662	1.00E-06	2.80E-05
G000157935_351299_354812	74.968	1.00E-06	2.80E-05
G000157935_189702_193215	68.722694	1.00E-06	2.80E-05
G000157935_1025794_1029307	68.65137	1.00E-06	2.80E-05
G000157935_1162801_1166314	67.70487	1.00E-06	2.80E-05
thdmi_cohort_G000157935_351299_354812	61.196087	1.00E-06	2.80E-05
G000157935_1208470_1211983	60.285686	1.00E-06	2.80E-05
G000157935_2532868_2536381	58.63104	1.00E-06	2.80E-05
G000157935_1647594_1651107	30.86908	1.00E-06	2.80E-05
G000157935_7026_10539	28.434813	1.00E-06	2.80E-05
G000157935_2817420_2820933	26.99588	1.00E-06	2.80E-05
G000157935_266987_270500	17.455702	1.00E-06	2.80E-05
G000157935_270500_274013	12.221665	3.00E-06	8.40E-05
bowel_movement_quality_G000157935_351299_354	10.751162	1.00E-06	2.80E-05
16s_with_prev	10.531625	1.50E-05	4.20E-04
antibiotic_history_G000157935_351299_354812	9.855121	1.00E-06	2.80E-05
types_of_plants_G000157935_351299_354812	8.156946	1.00E-06	2.80E-05
bowel_movement_frequency_G000157935_351299_	7.3015785	1.00E-06	2.80E-05
G000157935_1064437_1067950	4.222139	7.14E-03	2.00E-01
bowel_movement_quality	3.90906	3.10E-05	8.68E-04
bmi_cat	3.817884	1.98E-04	5.54E-03
age_cat	3.0832195	3.20E-05	8.96E-04
bowel_movement_frequency	2.7228851	2.23E-04	6.24E-03
antibiotic_history	2.4626591	4.16E-03	1.16E-01
types_of_plants	1.8151271	2.59E-02	7.25E-01

m-gnavus-raw (Figure 2j)

label_A	label_B	ks-statistic	ks-pvalue	corrected
control sample	creeping_fat	0.19047619	3.99E-01	1.00E+00
control sample	distant_mesenteric_fat	0.210526315	6.12E-01	1.00E+00
control sample	involved_mucosa	0.530612244	4.20E-06	1.18E-04
control sample	mesenteric_fat	0.329721362	7.18E-03	2.01E-01
control sample	subcutaneous_fat	0.107894736	9.53E-01	1.00E+00
control sample	uninvolved_mucosa	0.333333333	1.09E-02	3.06E-01
control sample	Monte Carlo unfocused (n=68)	0.253095975	7.12E-02	1.00E+00
creeping_fat	distant_mesenteric_fat	0.112781954	9.86E-01	1.00E+00
creeping_fat	involved_mucosa	0.401360544	8.92E-04	2.50E-02
creeping_fat	mesenteric_fat	0.235294117	9.27E-02	1.00E+00
creeping_fat	subcutaneous_fat	0.184523809	4.20E-01	1.00E+00
creeping_fat	uninvolved_mucosa	0.191876750	3.14E-01	1.00E+00
creeping_fat	Monte Carlo unfocused (n=68)	0.108543417	8.78E-01	1.00E+00
distant_mesenteric_fat	involved_mucosa	0.372717508	3.23E-02	9.04E-01
distant_mesenteric_fat	mesenteric_fat	0.213622291	4.36E-01	1.00E+00
distant_mesenteric_fat	subcutaneous_fat	0.228947368	4.27E-01	1.00E+00
distant_mesenteric_fat	uninvolved_mucosa	0.182662538	6.64E-01	1.00E+00
distant_mesenteric_fat	Monte Carlo unfocused (n=68)	0.117647058	9.66E-01	1.00E+00
involved_mucosa	mesenteric_fat	0.319927971	4.19E-03	1.17E-01
involved_mucosa	subcutaneous_fat	0.518877551	5.82E-06	1.63E-04
involved_mucosa	uninvolved_mucosa	0.326930772	6.49E-03	1.82E-01
involved_mucosa	Monte Carlo unfocused (n=68)	0.307623049	6.70E-03	1.88E-01
mesenteric_fat	subcutaneous_fat	0.373529411	1.19E-03	3.32E-02
mesenteric_fat	uninvolved_mucosa	0.205882352	1.50E-01	1.00E+00
mesenteric_fat	Monte Carlo unfocused (n=68)	0.161764705	3.38E-01	1.00E+00
subcutaneous_fat	uninvolved_mucosa	0.292156862	3.31E-02	9.27E-01
subcutaneous_fat	Monte Carlo unfocused (n=68)	0.251470588	6.76E-02	1.00E+00
uninvolved_mucosa	Monte Carlo unfocused (n=68)	0.107843137	8.56E-01	1.00E+00

Supplementary Table 3. Benchmarking results

Number of alignment	Time_micov(s)	Sort_bam(s)	Time_coverm(s)
10	5.1	0.0	22.5
100	1.5	0.0	20.2
1,000	1.6	0.0	23.1
10,000	1.8	0.1	24.5
100,000	2.3	0.8	25.2
1,000,000	6.1	7.7	27.6
10,000,000	50.3	89.1	40.2

Supplementary Note 1

The cumulative coverage approach we propose in micov bypasses the selection of a coverage breath threshold in detecting presence/absence of a particular microbial taxon in metagenomic samples. Here we use the creeping fat dataset to demonstrate the challenges of presence/absence based analysis in low biomass settings.

Figure 2j contains Kolmogorov-Smirnov test results comparing the cumulative coverage breadth of *Mediterraneibacter gnavus* among prospectively acquired, surgically resected, paired human mucosal and adipose tissue samples from patients with Crohn's disease. As these are tissue samples, they are anticipated to be low microbial biomass.

To conduct a "presence / absence" based statistical test using the same data, we first need to determine the coverage breadth threshold which defines the presence of *M. gnavus* in the sample, which is a critical parameter for such an analysis. We first plotted the number of samples with *M. gnavus* present at different coverage breadth thresholds (**Figure N1**). At thresholds higher than 78.8%, 0 out of 307 samples have *M. gnavus* present. Therefore, we limit our analysis to thresholds below 78.8% and approached this analysis using five different coverage thresholds 0.1%, 1%, 10%, 50%, and 70%. Note that the cumulative coverage plot achieves a higher coverage than any individual biological sample, which would occur if the genome (or a near relative) is present and being observed with approximately uniformly sampled short sequencing reads.

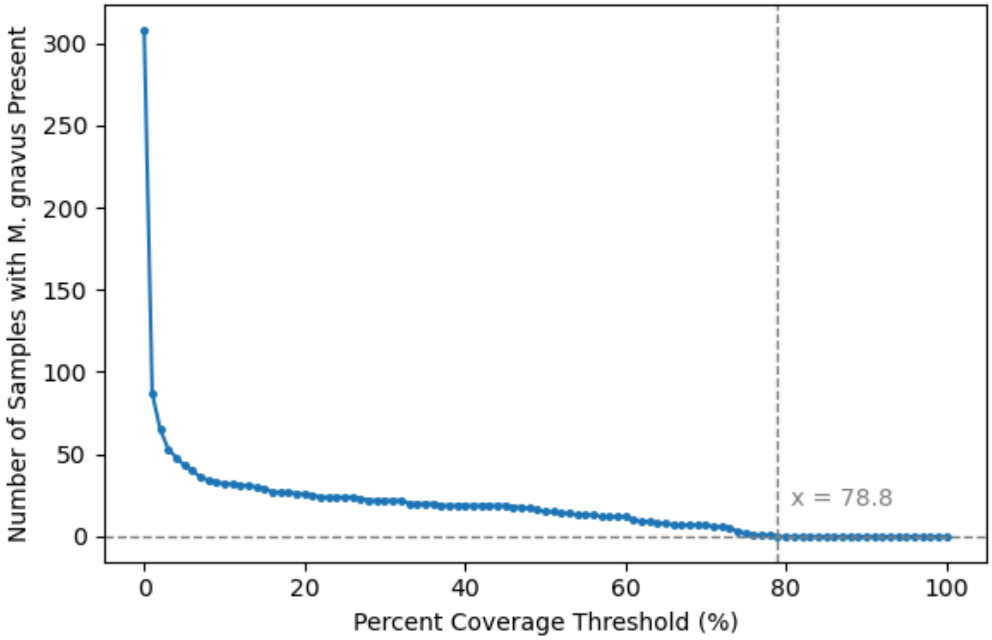

Figure N1: Number of samples with *M. gnavus* present at different coverage thresholds.

At each threshold, we classified samples as *M. gnavus* present or absent based on the threshold and plotted their distributions across tissue types. We then perform

pairwise Fisher's Exact Tests between them. *P*-values are corrected using the Benjamini-Hochberg method. Only significant comparisons are shown, ordered by increasing adjusted *p*-values.

At lower thresholds (0.1% and 1%), we observed a strong association of *M. gnavus* presence and involved mucosa compared to other tissue types including subcutaneous fat, negative controls, creeping fat, supporting results outlined in 2j. In addition, the comparison between involved mucosa and mesenteric fat is also significant (**Figure N2, N3**).

At medium thresholds (10% and 50%), significant differences remained between involved mucosa and the same tissue types, although the ranking of significant comparisons varied: at the 10% threshold, the most significant comparison was between involved mucosa and negative controls (**Figure N4**), whereas at the 50% threshold, it was observed between involved mucosa and mesenteric fat (**Figure N5**).

However, at the 70% threshold, *M. gnavus* was absent from the majority of samples, and no statistically significant associations were observed (**Figure N6**). These results highlight how the choice of coverage breadth threshold can dramatically influence outcomes in presence/absence analyses.

We note that determining an appropriate coverage threshold for presence/absence analysis using short reads is inherently challenging, and to the best of our knowledge, the state-of-the-art is to use an arbitrary value. Coverage breadth can be affected by factors such as genome abundance, genome size, and sequencing depth. In low-biomass settings, it is particularly difficult to distinguish between true low-abundance genomes and false positives, both of which often display low coverage.

The cumulative coverage approach we propose in micov and used in Figure 2i and 2j bypasses the selection of a coverage breath threshold, and considers the continuum of possible thresholds. It demonstrates sensitivity for genome detection and differentiation in low-biomass settings by leveraging the cumulative growth of coverage breadth across all samples (Figure 2i).

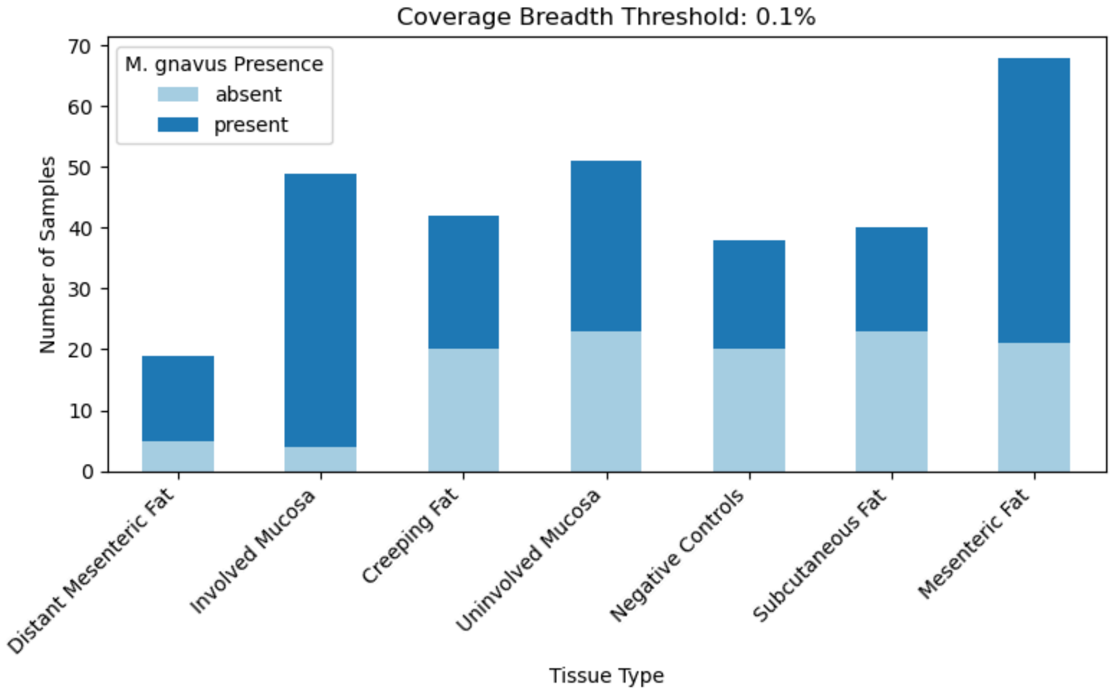

Tissue Type A	Tissue Type B	Counts A (present, absent)	Counts B (present, absent)	Odds Ratio	Raw p-value	Adjusted p-value (BH)	Significant (α=0.05)
Involved Mucosa	Subcutaneous Fat	[45, 4]	[17, 23]	15.220588	4.647749e-07	0.000010	True
Involved Mucosa	Negative Controls	[45, 4]	[18, 20]	12.500000	5.807967e-06	0.000061	True
Creeping Fat	Involved Mucosa	[22, 20]	[45, 4]	0.097778	2.756728e-05	0.000170	True
Involved Mucosa	Uninvolved Mucosa	[45, 4]	[28, 23]	9.241071	3.244555e-05	0.000170	True
Involved Mucosa	Mesenteric Fat	[45, 4]	[47, 21]	5.026596	3.001878e-03	0.012608	True
Mesenteric Fat	Subcutaneous Fat	[47, 21]	[17, 23]	3.028011	8.534418e-03	0.029870	True

Figure N2: Presence/Absence of *M. gnavus* across tissue types. Coverage breadth threshold for organism presence is 0.1%.

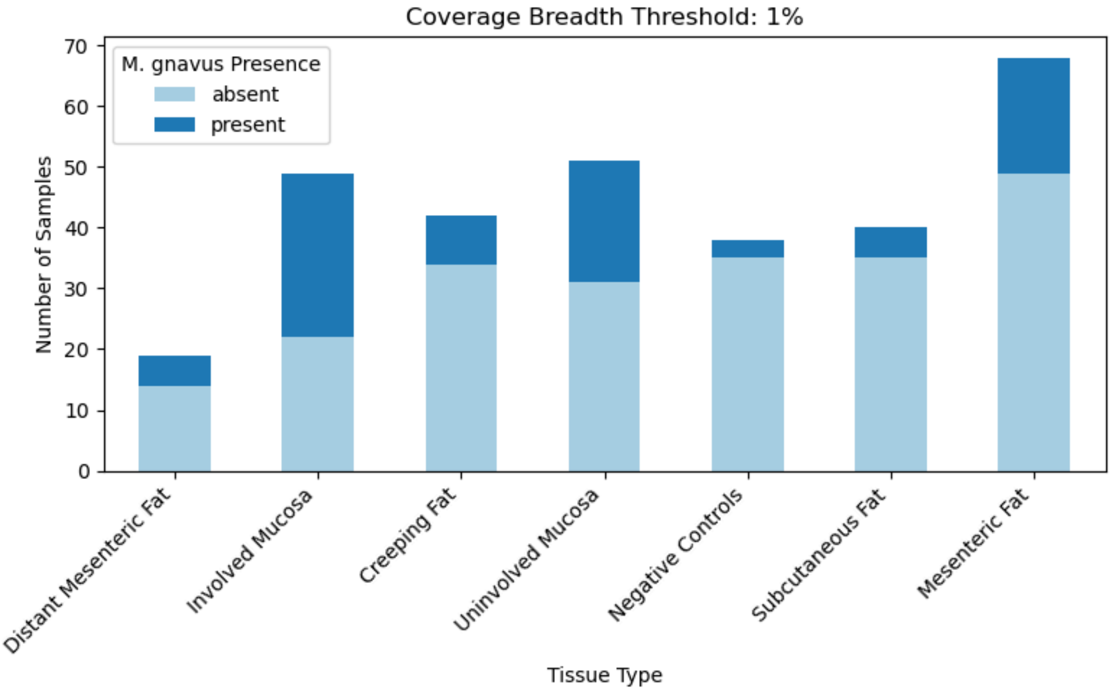

Tissue Type A	Tissue Type B	Counts A (present, absent)	Counts B (present, absent)	Odds Ratio	Raw p-value	Adjusted p-value (BH)	Significant (α=0.05)
Involved Mucosa	Negative Controls	[27, 22]	[3, 35]	14.318182	0.000003	0.000064	True
Involved Mucosa	Subcutaneous Fat	[27, 22]	[5, 35]	8.590909	0.000044	0.000466	True
Creeping Fat	Involved Mucosa	[8, 34]	[27, 22]	0.191721	0.000520	0.003640	True
Negative Controls	Uninvolved Mucosa	[3, 35]	[20, 31]	0.132857	0.001149	0.006030	True
Involved Mucosa	Mesenteric Fat	[27, 22]	[19, 49]	3.165072	0.003998	0.016794	True
Subcutaneous Fat	Uninvolved Mucosa	[5, 35]	[20, 31]	0.221429	0.004936	0.017275	True

Figure N3: Presence/Absence of *M. gnavus* across tissue types. Coverage breadth threshold for organism presence is 1%.

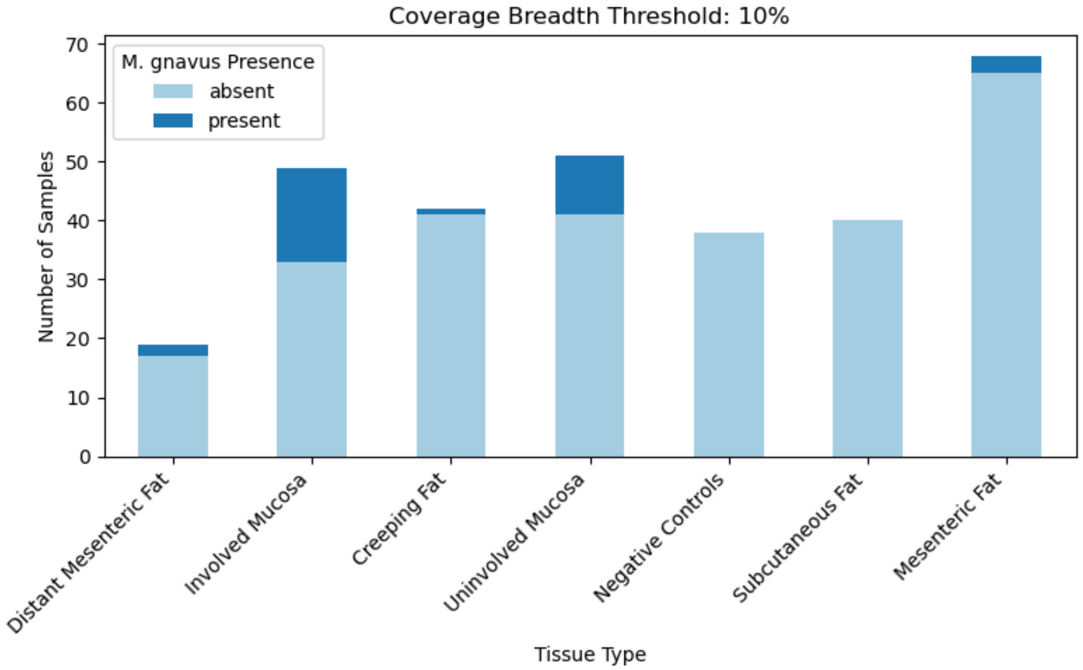

Tissue Type A	Tissue Type B	Counts A (present, absent)	Counts B (present, absent)	Odds Ratio	Raw p-value	Adjusted p-value (BH)	Significant (α=0.05)
Involved Mucosa	Negative Controls	[16, 33]	[0, 38]	inf	0.000035	0.000366	True
Involved Mucosa	Subcutaneous Fat	[16, 33]	[0, 40]	inf	0.000030	0.000366	True
Involved Mucosa	Mesenteric Fat	[16, 33]	[3, 65]	10.505051	0.000063	0.000439	True
Creeping Fat	Involved Mucosa	[1, 41]	[16, 33]	0.050305	0.000236	0.001241	True
Subcutaneous Fat	Uninvolved Mucosa	[0, 40]	[10, 41]	0.000000	0.002120	0.008904	True
Negative Controls	Uninvolved Mucosa	[0, 38]	[10, 41]	0.000000	0.004241	0.014842	True
Creeping Fat	Uninvolved Mucosa	[1, 41]	[10, 41]	0.100000	0.010886	0.032657	True
Mesenteric Fat	Uninvolved Mucosa	[3, 65]	[10, 41]	0.189231	0.014890	0.039086	True

Figure N4: Presence/Absence of *M. gnavus* across tissue types. Coverage breadth threshold for organism presence is 10%.

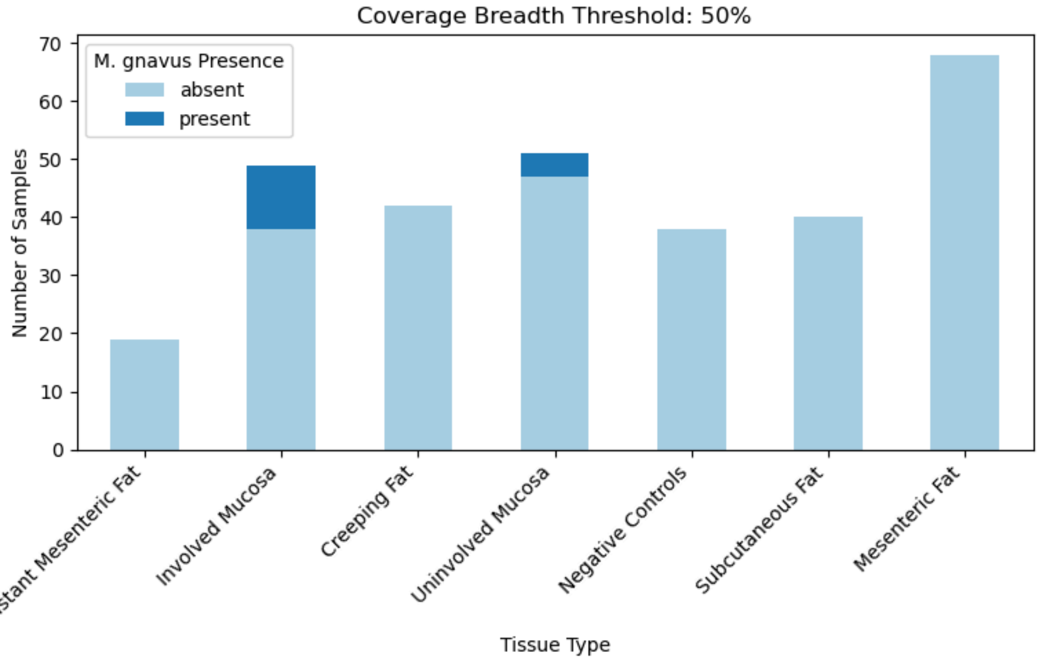

Tissue Type A	Tissue Type B	Counts A (present, absent)	Counts B (present, absent)	Odds Ratio	Raw p-value	Adjusted p-value (BH)	Significant (α=0.05)
Involved Mucosa	Mesenteric Fat	[11, 38]	[0, 68]	inf	0.000034	0.000705	True
Creeping Fat	Involved Mucosa	[0, 42]	[11, 38]	0.0	0.000706	0.006028	True
Involved Mucosa	Subcutaneous Fat	[11, 38]	[0, 40]	inf	0.000861	0.006028	True
Involved Mucosa	Negative Controls	[11, 38]	[0, 38]	inf	0.001910	0.010030	True

Figure N5: Presence/Absence of *M. gnavus* across tissue types. Coverage breadth threshold for organism presence is 50%.

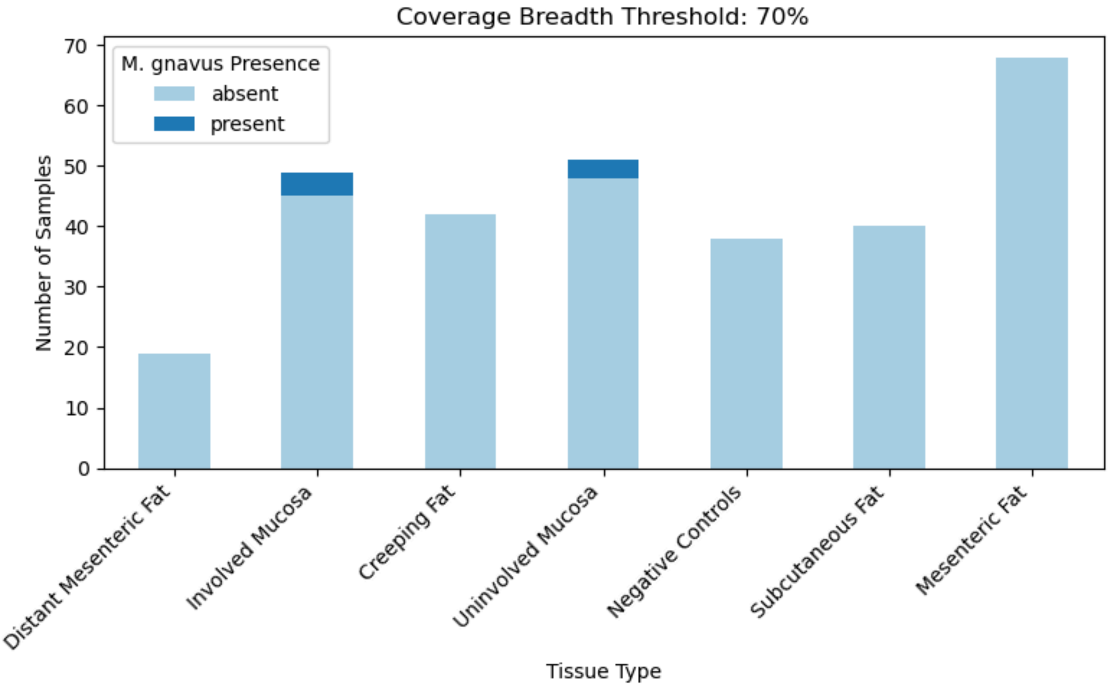

Tissue Type A	Tissue Type B	Counts A (present, absent)	Counts B (present, absent)	Odds Ratio	Raw p-value	Adjusted p-value (BH)	Significant (α=0.05)

Figure N6: Presence/Absence of *M. gnavus* across tissue types. Coverage breadth threshold for organism presence is 70%.
