# Supplementary material for: Calculating fast differential genome coverages among metagenomic sources using micov
Source: Commun Biol. 2025 Nov 20;8:1624. doi: 10.1038/s42003-025-09007-6 (PMC12635244; doi:10.1038/s42003-025-09007-6)
Supplement: Supplementary file 2 — Description of Additional Supplementary Materials [file 42003_2025_9007_MOESM2_ESM.pdf]

Description of Additional Supplementary Files

File name: Supplementary Data 1

Description: specific experiment accessions
