# Supplementary material for: Calculating fast differential genome coverages among metagenomic sources using micov
Source: Commun Biol. 2025 Nov 20;8:1624. doi: 10.1038/s42003-025-09007-6 (PMC12635244; doi:10.1038/s42003-025-09007-6)
Supplement: Supplementary file 4 — Reporting Summary [file 42003_2025_9007_MOESM4_ESM.pdf]

nature portfolio

Corresponding author(s): Rob Knight

Last updated by author(s): Sep 1, 2025

Reporting Summary

Nature Portfolio wishes to improve the reproducibility of the work that we publish. This form provides structure for consistency and transparency in reporting. For further information on Nature Portfolio policies, see our Editorial Policies and the Editorial Policy Checklist.

Statistics

For all statistical analyses, confirm that the following items are present in the figure legend, table legend, main text, or Methods section.

n/a	Confirmed	
☐	☒	The exact sample size (*n*) for each experimental group/condition, given as a discrete number and unit of measurement
☐	☒	A statement on whether measurements were taken from distinct samples or whether the same sample was measured repeatedly
☐	☒	The statistical test(s) used AND whether they are one- or two-sided<br>*Only common tests should be described solely by name; describe more complex techniques in the Methods section.*
☐	☒	A description of all covariates tested
☐	☒	A description of any assumptions or corrections, such as tests of normality and adjustment for multiple comparisons
☐	☒	A full description of the statistical parameters including central tendency (e.g. means) or other basic estimates (e.g. regression coefficient) AND variation (e.g. standard deviation) or associated estimates of uncertainty (e.g. confidence intervals)
☐	☒	For null hypothesis testing, the test statistic (e.g. *F*, *t*, *r*) with confidence intervals, effect sizes, degrees of freedom and *P* value noted<br>*Give P values as exact values whenever suitable.*
☒	☐	For Bayesian analysis, information on the choice of priors and Markov chain Monte Carlo settings
☐	☒	For hierarchical and complex designs, identification of the appropriate level for tests and full reporting of outcomes
☐	☒	Estimates of effect sizes (e.g. Cohen's *d*, Pearson's *r*), indicating how they were calculated

Our web collection on statistics for biologists contains articles on many of the points above.

Software and code

Policy information about availability of computer code

Data collection	Data was privately processed using Qitta.
Data analysis	Python software, custom algorithms written in Python (https://github.com/biocore/micov)

For manuscripts utilizing custom algorithms or software that are central to the research but not yet described in published literature, software must be made available to editors and reviewers. We strongly encourage code deposition in a community repository (e.g. GitHub). See the Nature Portfolio guidelines for submitting code & software for further information.

Data

Policy information about availability of data

All manuscripts must include a data availability statement. This statement should provide the following information, where applicable:

- Accession codes, unique identifiers, or web links for publicly available datasets
- A description of any restrictions on data availability
- For clinical datasets or third party data, please ensure that the statement adheres to our policy

All data will be publicly available at the time of publication. Access details will be provided in the manuscript under the Data Availability and Code Availability sections.

Research involving human participants, their data, or biological material

Policy information about studies with human participants or human data. See also policy information about sex, gender (identity/presentation), and sexual orientation and race, ethnicity and racism.

Reporting on sex and gender	New datasets involved in this manuscript include the EPEC and visceral fat datasets, of which only the visceral fat dataset involved human participants. We answer the following questions with regards to the visceral fat dataset. We do not report on sex and gender.
Reporting on race, ethnicity, or other socially relevant groupings	We do not report on race, ethnicity, or other socially relevant groupings.
Population characteristics	We do not report on population characteristics.
Recruitment	We do not report on recruitment.
Ethics oversight	This study is a software paper and does not have an ethics board specific to this study. Tissue sampling methods and human subjects approval are previously published.

Note that full information on the approval of the study protocol must also be provided in the manuscript.

Field-specific reporting

Please select the one below that is the best fit for your research. If you are not sure, read the appropriate sections before making your selection.

☒ Life sciences ☐ Behavioural & social sciences ☐ Ecological, evolutionary & environmental sciences

For a reference copy of the document with all sections, see nature.com/documents/nr-reporting-summary-flat.pdf

Life sciences study design

All studies must disclose on these points even when the disclosure is negative.

Sample size	Whenever possible, we utilized all available data to maximize the sample size.
Data exclusions	Data exclusion was not relevant to the metagenomics datasets used in the study. We included data from a variety of sample types and across various levels of biomass.
Replication	Replication was performed by leveraging Qiita to run reproducible metagenomics workflows.
Randomization	Randomization was not relevant to the metagenomics datasets used in the study as this study focuses solely on software development and implementation.
Blinding	Blinding was not relevant to the metagenomics datasets used in the study as this study focuses solely on software development and implementation.

Reporting for specific materials, systems and methods

We require information from authors about some types of materials, experimental systems and methods used in many studies. Here, indicate whether each material, system or method listed is relevant to your study. If you are not sure if a list item applies to your research, read the appropriate section before selecting a response.

Materials & experimental systems

n/a	Involved in the study	
☒	☐	Antibodies
☒	☐	Eukaryotic cell lines
☒	☐	Palaeontology and archaeology
☒	☐	Animals and other organisms
☒	☐	Clinical data
☒	☐	Dual use research of concern
☒	☐	Plants

Methods

n/a	Involved in the study	
☒	☐	ChIP-seq
☒	☐	Flow cytometry
☒	☐	MRI-based neuroimaging

Plants

Seed stocks	Not plants involved.
Novel plant genotypes	Not plants involved.
Authentication	Not plants involved.
